# Heart rate variability: a predictor of cognitive decline

**DOI:** 10.18632/aging.204715

**Published:** 2023-09-20

**Authors:** Paola Nicolini, Tiziano Lucchi, Marco Vicenzi

**Affiliations:** 1 Fondazione IRCCS Ca’ Granda Ospedale Maggiore Policlinico, Geriatric Unit, Internal Medicine Department, Milan, Italy; 2 Dyspnea Lab, Department of Clinical Sciences and Community Health, University of Milan, Milan, Italy; 3 Fondazione IRCCS Ca' Granda Ospedale Maggiore Policlinico, Department of Cardio-Thoracic-Vascular Diseases, Milan, Italy

**Keywords:** heart rate variability, mild cognitive impairment, cognitive decline, autonomic nervous system, longitudinal studies

Heart rate variability (HRV) refers to the fluctuations in the time intervals between consecutive heartbeats on an electrocardiographic (ECG) recording. It reflects the influence on sinus node activity of the sympathetic and parasympathetic branches of the autonomic nervous system and is a reliable, non-invasive and inexpensive index of autonomic function [1].

There is increasing cross-sectional evidence of an association between HRV and cognition, in subjects with neurodegenerative disorders [2, 3] as well as in healthy individuals across the age spectrum [3, 4], providing support to the potential role of HRV as a biomarker of cognitive functioning [2–4]. However, very few longitudinal studies have investigated HRV as a predictor of cognition, and none have considered participants with a definite baseline diagnosis of cognitive status, i.e. normal cognition (NC), Mild Cognitive Impairment (MCI) and dementia. Also, the available longitudinal studies sometimes employ ultra-short-term (< 5 minutes) HRV with uncertain validity, mostly lack provocative tests that enhance sensitivity to autonomic dysfunction, occasionally only gauge cognition at follow-up, and invariably use limited cognitive testing with little or no assessment of episodic memory [5].

Among the several possible links between HRV and cognition, the Central Autonomic Network (CAN) is attracting growing attention [2–4, 6]. The CAN is an intricate network of brain regions implicated both in cognitive processing and in the regulation of cardiovascular function via projections to the preganglionic neurons of the sympathetic and parasympathetic nervous system, and it represents the neuroanatomical substrate of the heart-brain axis [3, 6]. There is accumulating recognition that different components of the CAN are engaged in different cognitive functions and in sympathetic versus parasympathetic autonomic control, with the hippocampus, insula and locus coeruleus involved in episodic memory and sympathetic outflow, and the prefrontal cortex involved in executive functioning and parasympathetic outflow [5, 6]. Accordingly, in the psychophysiological literature, better episodic memory has been reported to correlate with HRV indices pointing to higher sympathetic activity, and better executive functioning has been reported, more widely and consistently, to correlate with HRV indices denoting higher parasympathetic activity [2–6].

The paucity of longitudinal research on HRV and cognition, coupled with emerging data on the site-specific functions of the CAN, set the premises for a recent study by our group [5]. We enrolled older subjects (aged ≥ 65) with a baseline diagnosis of NC or MCI whose HRV was concurrently evaluated on 5-minute ECG recordings in resting conditions and during a sympathetic (i.e. active standing) and parasympathetic (i.e. paced breathing at 12 breaths/min) challenge. The dynamic response to the challenge was computed as ∆ HRV (i.e. HRV during the challenge minus HRV at rest) and focused on the normalized low frequency power (LFn) and the low frequency power to high frequency power (LF/HF) ratio which are known to increase during active standing (indicating sympathetic activation/parasympathetic withdrawal) and to decrease during paced breathing (indicating parasympathetic activation/sympathetic withdrawal). Cognitive functioning was assessed at baseline and at a 3-year follow-up by means of an extensive neuropsychological battery covering numerous executive functions and both verbal and visual episodic memory. After accounting for loss to follow-up (n = 9) the sample was composed of 71 subjects (n = 37 NC and n = 34 MCI).

We found that in the MCI group a greater response to a sympathetic challenge (i.e. more positive ∆ LFn and ∆ LF/HF in response to active standing) predicted a greater decline in episodic memory, whereas a greater response to a parasympathetic challenge (i.e. more negative ∆ LFn and ∆ LF/HF in response to paced breathing) predicted a lesser decline in executive functioning. While the second finding was anticipated, the first finding was in the opposite direction than expected. Although we speculated this may be related to the inverse U-shaped curve of brain activation versus brain damage, by which subjects with higher brain activity are closer to the apex of the curve beyond which cognitive decline is steeper, the mechanism underlying such unforeseen result remains to be elucidated.

These findings extend our previous cross-sectional work on autonomic dysfunction in all-type [7] and specific-type (amnestic/non amnestic) MCI [8]. They will need to be corroborated and expanded upon by future research conducted on larger cohorts, also encompassing functional neuroimaging of the CAN and other non-HRV indices of sympathetic activity (e.g. the impedance-derived systolic time intervals), differentiating MCI subtypes, and incorporating a follow-up HRV measurement to explore the yet unaddressed question of whether changes in cognition co-occur with changes in HRV.

To the best of our knowledge, our study is the first to show that HRV is a predictor of cognitive change in subjects with MCI, and it highlights a distinct effect of the two limbs of the autonomic nervous system on the domains of episodic memory and executive functioning. These data contribute insight to the likely complex and nuanced prospective relationship between HRV and cognition, and suggest that, within the heterogenous cognitive trajectories of MCI, the HRV response to a physical challenge may help identify subjects at higher risk of cognitive decline to whom interventions should be targeted.

## References

[1] Malik M, et al. European Heart Journal. 1996; 17: 354–81. 10.1093/oxfordjournals.eurheartj.a014868 8737210

[2] Liu KY, et al. Ageing Res Rev. 2022; 73:101539. 10.1016/j.arr.2021.101539 34883203 PMC8783051

[3] Arakaki X, et al. Front Neurosci. 2023; 17:1055445. 10.3389/fnins.2023.1055445 36937689 PMC10014754

[4] Forte G, et al. Front Neurosci. 2019; 13:710. 10.3389/fnins.2019.00710 31354419 PMC6637318

[5] Nicolini P, et al. Front Aging Neurosci. 2022; 14:886023. 10.3389/fnagi.2022.886023 36185491 PMC9520613

[6] Thayer JF, et al. Ann Behav Med. 2009; 37:141–53. 10.1007/s12160-009-9101-z 19424767

[7] Nicolini P, et al. PLoS One. 2014; 9:e96656. 10.1371/journal.pone.0096656 24801520 PMC4011966

[8] Nicolini P, et al. Sci Rep. 2020; 10:11661. 10.1038/s41598-020-68131-x 32669640 PMC7363846

